# Profile measurement adopting binocular active vision with normalization object of vector orthogonality

**DOI:** 10.1038/s41598-019-41341-8

**Published:** 2019-04-02

**Authors:** Guan Xu, Junyi Chen, Xiaotao Li, Jian Su

**Affiliations:** 1 School of Transportation, Nanling Campus, Jilin University, Renmin Str. 5988#, Changchun, China; 2 School of Mechanical and Aerospace Engineering, Nanling Campus, Jilin University, Renmin Str. 5988#, Changchun, China

## Abstract

Active-vision-based measurement plays an important role in the profile inspection study. The binocular vision, a passive vision, is employed in the active vision system to contribute the benefits of them. The laser plane is calibrated by two 2D targets without texture initially. Then, an L target with feature points is designed to construct the orthogonality object of two vectors. In order to accurately model the binocular-active-vision system, the feature points on the L target are built by two cameras and parameterized by the laser plane. Different from the optimization methods on the basis of the distance object, the laser plane is further refined by the distance-angle object. Thus, an optimization function is created considering both the norms and angles of the vectors. However, the scale of the distance is diverse from the scale of the angle. Therefore, the optimization function is enhanced by the normalization process to balance the different scales. The comparison experiments show that the binocular active vision with the normalization object of vector orthogonality achieves the decreasing distance errors of 25%, 22%, 13% and 4%, as well as the decreasing angle errors of 23%, 20%, 14% and 4%, which indicates an accurate measurement to reconstruct the object profile.

## Introduction

Profile measurement is one of the most important techniques in the optical inspection and vision^1–4^. Recently, information acquirement of the 3D profile is popular in the research fields of the automotive industry^5^, the robot vision^6^, the medical diagnosis^7^, the reverse engineering^8^, etc. However, the camera only generates 2D images without depth information, which restricts us to acquire and perceive the profile of object in the real world.

Many advances to solve the problem of profile measurement are reported in recent years^9^. The developments of the profile measurement can be classified into two main parts according to the way for acquiring the third dimensional information. The first kind of methods contributes the third dimensional information by the other camera, i.e. the binocular vision or the stereo vision. Faugerass O.^10^ provides the classical work of computational stereo in image-based reconstruction. The properties of the stereo vision, including epipolar geometry, are concluded according to the camera projective model. The fundamental matrix of the stereo vision is also estimated in the literature. Grimson W. E. L.^11,12^ presents an unsupervised learning method to model activities and cluster trajectories through a multi-camera system. The framework groups the trajectories corresponding to the same activities. Then the paths are extracted from the views of the cameras. Thirdly, the abnormal activities are detected by the probabilistic model. The activity model is suitable for recognizing the scene structures. The improvements are the uncalibrated cameras in the method and the activity learning without supervision. The framework focuses on the activity model and scene structure recognition. However, only positions and directions are extracted as features of trajectories. The accurate profile and size of object are not reconstructed in the paper. Ren Z.^13^ proposes a 3D measurement method of small mechanical parts with binocular vision. The complicated noisy impact is considered in the test. The nonlinear features are precisely extracted by the multiscale decomposition of images and virtual chains. The 3D curves are reconstructed by the projections of two cameras. Ambrosch K.^14^ provides the hardware for the real-time stereo vision. A novel algorithm for the absolute differences and census transform^15^ is proposed for stereo matching. Wang Z.^16^ introduces a calibration method for the parameters of binocular vision system. The test is performed by the small target in the large field of view. The re-projection errors and Zhang’s method^17^ are employed to conduct the optimization solution of the parameters. Although the binocular vison is simple to be realized, the feature matching is still a complex problem for the object without texture. Therefore, the binocular vision is widely mentioned in 3D pose estimation^18,19^, visual navigation^20^, etc.

The second kind of profile measurement methods is active vision, which generates the third dimensional information by the structured light. The light pattern in the active vision includes points^21^, lines^22^ and gratings^23,24^. Xu G.^25^ outlines a model to reconstruct the 3D laser projection point on the measured object. A point laser projector is combined to a planar target, where the projection invariants are derived. Rodríguez J. A. M.^22^ presents a 3D vision method using the mobile camera and the laser line. An approximation network is constructed to calibrate the scanning system. Grating is the prevalent structured light in the active vision. Chen X.^26^ constructs a camera-projector system. Four sinusoidal gratings are projected on the measured object. The fourth order radial and tangential lens distortion are considered in the system calibration model. The active vision facilitates the profile measurement as the structured light is an idea mark on the object. Therefore, the active vision overcomes the matching problem of the object without texture. Chen, H.^27^ presents an improved calibration method to increase the calibration accuracy of the camera and the projector simultaneously. The previous works also cover the studies on the combination of the active vision and the binocular vision^28^. As the single camera has the problem of camera occlusion, Zhang S.^29^ develops a shape measurement method based on one grating projector and two cameras. The system model and 3D data registration with the iterative closest-point algorithm are described in the method. Vilaça J. L.^30^ reports a laser scanning system with two cameras to profile the 3D object surface. The two cameras achieve the laser line detection for the calibration of structured light. As the grating pattern tends to be impacted by the illumination in the environment, the measurement on the basis of laser line is appropriate for the accurate non-contact measurement. Jin Z. S.^31^ proposes a reconstruction method for the gas metal arc welding (GMAW) pool. The vision measurement system consists of two cameras and a laser generator. The point projection is constructed by the pinhole model. The two cameras and the laser plane are calibrated respectively. The method provides the 3D reconstruction result of weld pool surface. Nevertheless, the epipolar geometry, which relates the points on one camera and the lines on the other camera, is not mentioned in the reconstruction. Furthermore, only the closed form solutions are modeled in the paper. The optimization process based on the real linear dimension and angular dimension is also not reported in the method.

A binocular active vision approach is proposed to achieve the profile measurement. In the traditional research^15^, two main ideas are reported in the literatures. The first is based on the 2D re-projection errors, which are generated from the image and parameterized by the internal and external matrices of the active vision system. Obviously, the 3D reconstruction accuracy is not the optimization objective of the idea. Therefore, it is unsuitable for 3D profile reconstruction. The other approach employs the 3D reconstruction errors. The 3D distance is reconstructed by the parameters of the active vision system. The measurement accuracy of the linear dimension is improved in the optimization process. However, the measurement accuracy of the angular dimension is not considered in the optimization. In order to simultaneously improve the measurement accuracy of the real linear dimension and angular dimension, we focus on the orthogonality of vectors that are generated from an L target. Geometrically, two vectors are derived from the feature points on the L target. The norms of the vectors and the angle between the vectors are parameterized by the laser plane coordinate, the internal and external matrices of the cameras. According to the known angle and norms of the vectors on the L target, the angle reconstruction errors and the distance reconstruction errors are synthetically modeled in the optimization objective function. In the study, the measurement accuracy is enhanced by the optimization process. By this method, the distance and angle are both considered in the optimization object. Moreover, the different scales of the distance and angle are normalized to perform an accurate measurement. The rest paper is divided to three parts: Section 2 describes the solution model of the binocular active vision with the normalization object of the vector orthogonality. Then, Section 3 conducts the comprehensive comparison experiments to verify the method. Section 4 concludes the paper.

## Methods

**Figure 1:**
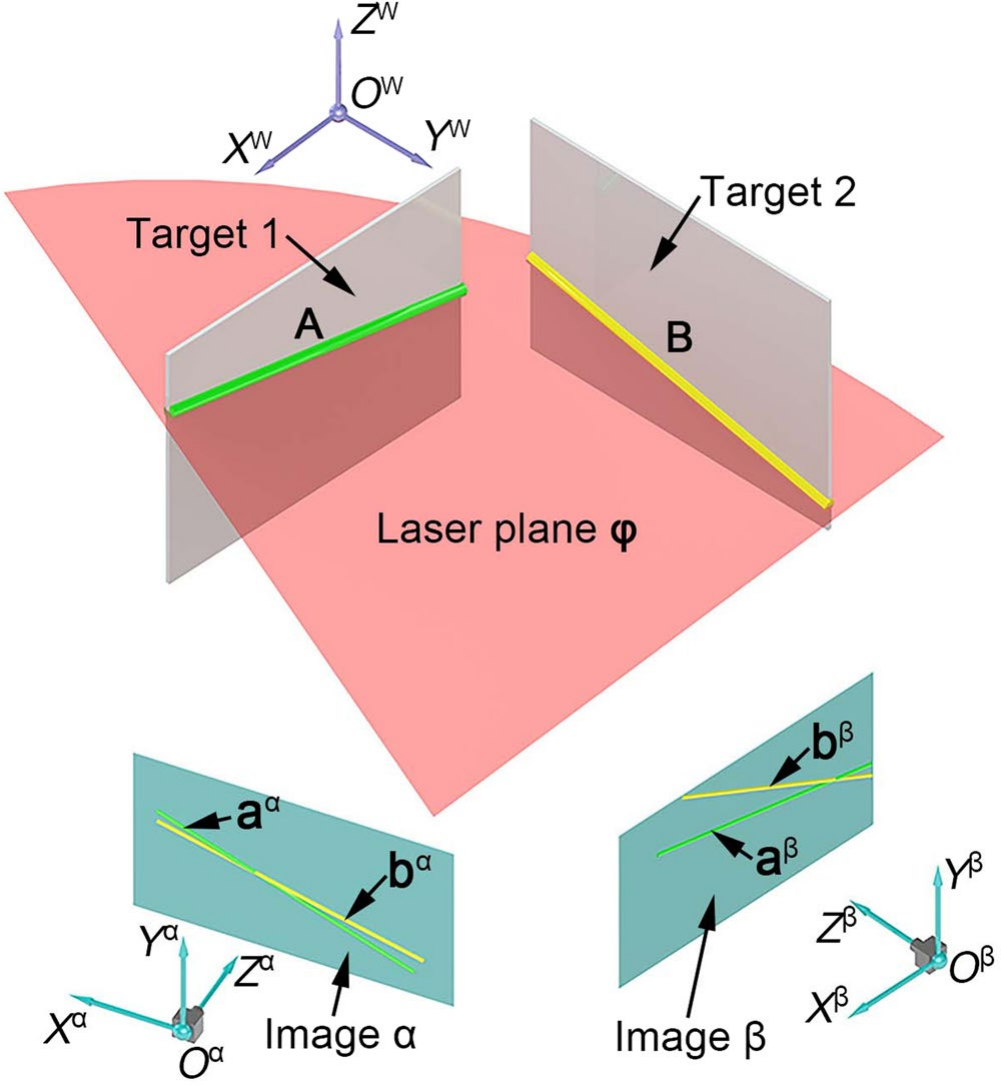
Initial calibration of the laser plane generated from two intersections between two targets and the laser plane in binocular vision.

The measurement model is illustrated in Fig. 1. Two targets without texture are designed and positioned in the view-field of the binocular vision system. *O*^α^-*X*^α^*Y*^α^*Z*^α^ and *O*^β^-*X*^β^*Y*^β^*Z*^β^ are the camera coordinate systems of the left and right cameras. *O*^W^-*X*^W^*Y*^W^*Z*^W^ is the world coordinate system. A laser plane is projected to the two targets. There are two intersection lines on the targets. Then the two cameras capture the laser intersection lines. The projections of the intersection lines are generated on the image planes of the two cameras.

The cameras are calibrated by the well-known direct linear transform (DLT) method^32^. The projection planes that are determined by the optical centers and projection laser lines are^33^

1 $${{\boldsymbol{\phi }}}^{{\rm{\alpha }},{\rm{{\rm I}}}}={({{\bf{P}}}^{{\rm{\alpha }}})}^{{\rm{T}}}{{\bf{a}}}^{{\rm{\alpha }}}$$

2 $${{\boldsymbol{\phi }}}^{{\rm{\alpha }},{\rm{{\rm I}}}{\rm{{\rm I}}}}={({{\bf{P}}}^{{\rm{\alpha }}})}^{{\rm{T}}}{{\bf{b}}}^{{\rm{\alpha }}}$$

3 $${{\boldsymbol{\phi }}}^{{\rm{\beta }},{\rm{{\rm I}}}}={({{\bf{P}}}^{{\rm{\beta }}})}^{{\rm{T}}}{{\bf{a}}}^{{\rm{\beta }}}$$

4 $${{\boldsymbol{\phi }}}^{{\rm{\beta }},{\rm{{\rm I}}}{\rm{{\rm I}}}}={({{\bf{P}}}^{{\rm{\beta }}})}^{{\rm{T}}}{{\bf{b}}}^{{\rm{\beta }}}$$

where **φ**^α,I^, **φ**^α,II^, **φ**^β,I^, **φ**^β,II^ are the projection planes. ${{\bf{P}}}^{{\rm{\alpha }}}={[{p}_{km}^{{\rm{\alpha }}}]}_{3\times 4}$, ${{\bf{P}}}^{{\rm{\beta }}}={[{p}_{km}^{{\rm{\beta }}}]}_{3\times 4}$ are the projection matrices of two cameras. **a**^α^, **b**^α^, **a**^β^, **b**^β^ are the projections of the laser lines. The superscripts α, β represent the left and right cameras. The superscripts I, II indicate the left and right targets.

The Plücker matrices of the intersection lines on the targets are^33^

5 $${{\bf{A}}}^{\ast }=({{\boldsymbol{\phi }}}^{{\rm{\alpha }},{\rm{{\rm I}}}})\,{({{\boldsymbol{\phi }}}^{{\rm{\beta }},{\rm{{\rm I}}}})}^{{\rm{T}}}-({{\boldsymbol{\phi }}}^{{\rm{\beta }},{\rm{{\rm I}}}})\,{({{\boldsymbol{\phi }}}^{{\rm{\alpha }},{\rm{{\rm I}}}})}^{{\rm{T}}}=[\begin{array}{cccc}0 & {({a}_{12})}^{\ast } & {({a}_{13})}^{\ast } & {({a}_{14})}^{\ast }\\ {({a}_{21})}^{\ast } & 0 & {({a}_{23})}^{\ast } & {({a}_{24})}^{\ast }\\ {({a}_{31})}^{\ast } & {({a}_{32})}^{\ast } & 0 & {({a}_{34})}^{\ast }\\ {({a}_{41})}^{\ast } & {({a}_{42})}^{\ast } & {({a}_{43})}^{\ast } & 0\end{array}]$$

6 $${{\bf{B}}}^{\ast }=({{\boldsymbol{\phi }}}^{{\rm{\alpha }},{\rm{{\rm I}}}{\rm{{\rm I}}}})\,{({{\boldsymbol{\phi }}}^{{\rm{\beta }},{\rm{{\rm I}}}{\rm{{\rm I}}}})}^{{\rm{T}}}-({{\boldsymbol{\phi }}}^{{\rm{\beta }},{\rm{{\rm I}}}{\rm{{\rm I}}}})\,{({{\boldsymbol{\phi }}}^{{\rm{\alpha }},{\rm{{\rm I}}}{\rm{{\rm I}}}})}^{{\rm{T}}}=[\begin{array}{cccc}0 & {({b}_{12})}^{\ast } & {({b}_{13})}^{\ast } & {({b}_{14})}^{\ast }\\ {({b}_{21})}^{\ast } & 0 & {({b}_{23})}^{\ast } & {({b}_{24})}^{\ast }\\ {({b}_{31})}^{\ast } & {({b}_{32})}^{\ast } & 0 & {({b}_{34})}^{\ast }\\ {({b}_{41})}^{\ast } & {({b}_{42})}^{\ast } & {({b}_{43})}^{\ast } & 0\end{array}]$$

where A^*^, B^*^ are the plane-based Plücker matrices of the intersection lines on the left and right targets, respectively.

**Figure 2:**
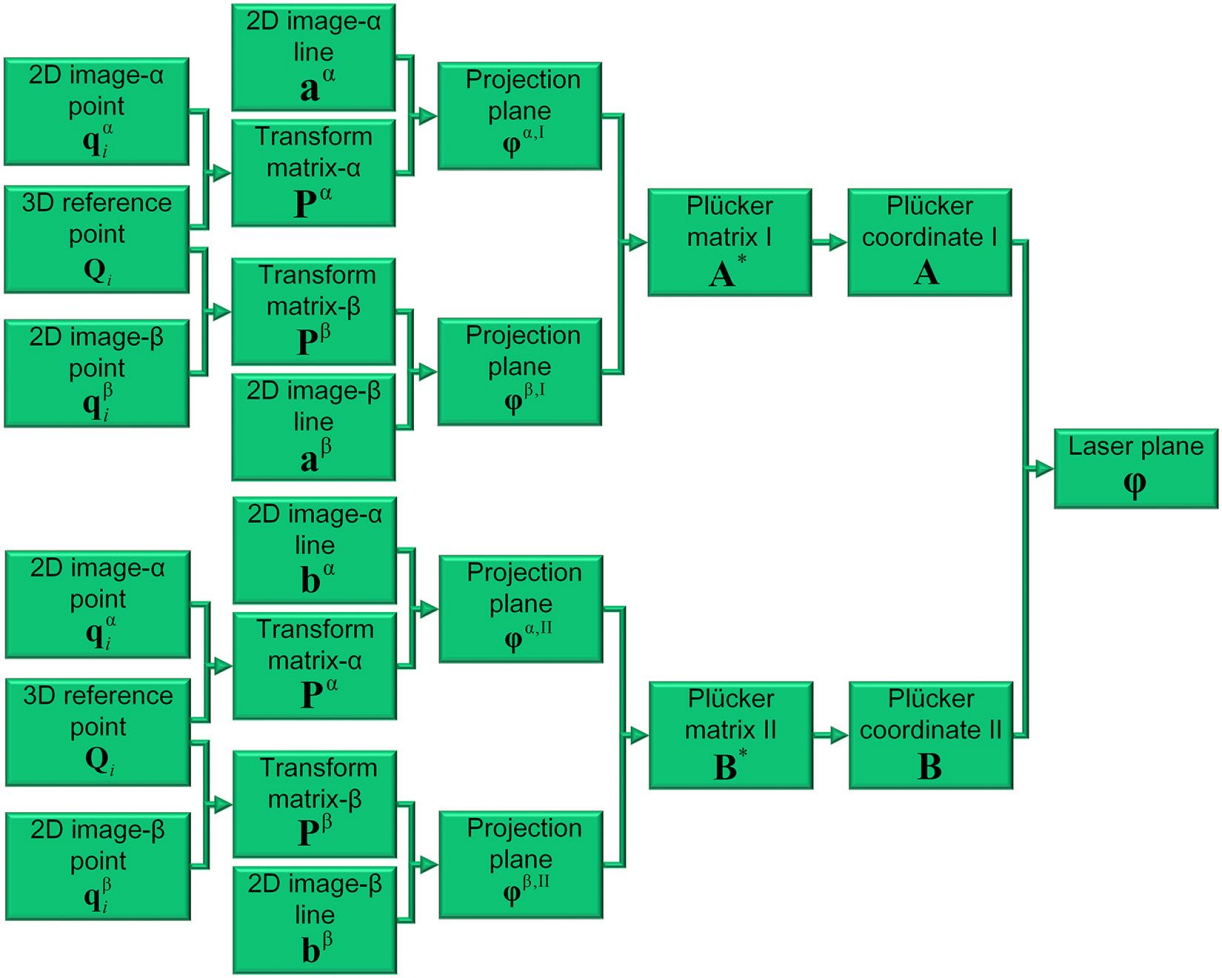
Solution process of the initial value of the laser plane.

The plane-based Plücker matrices A^*^, B^*^ are further transformed to the dual matrices **A**, **B** by the Graßmann–Plücker relation^32^. A laser plane can be determined by two laser lines **A**, **B**^34^. Thus, the laser plane satisfies

7 $${[{{\bf{A}}}^{{\rm{T}}}{{\bf{B}}}^{{\rm{T}}}]}^{{\rm{T}}}\,{\boldsymbol{\phi }}={\bf{0}}$$

where **φ** is the laser plane of the binocular-active-vision system. The solution process of the initial value of the laser plane is interpreted in Fig. 2.

**Figure 3:**
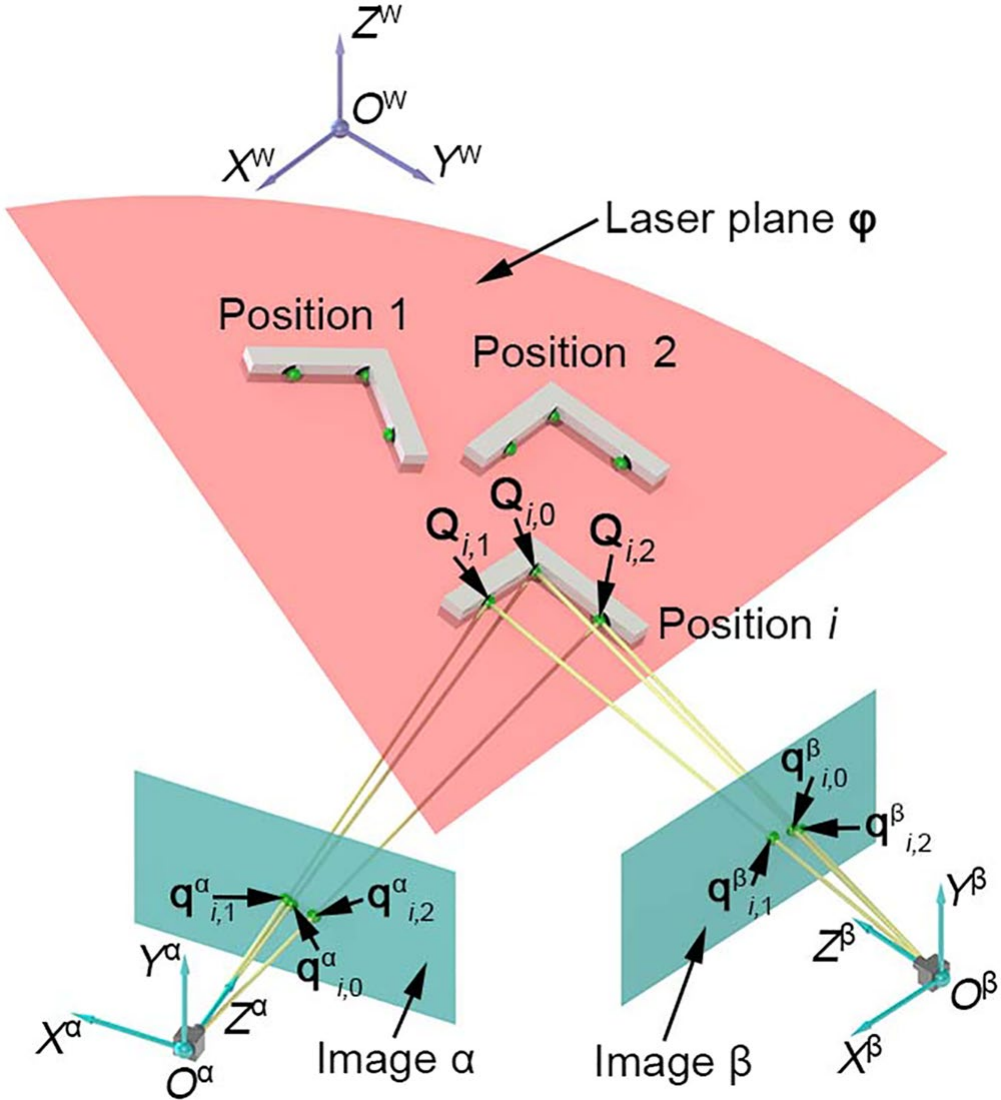
Optimization calibration of the laser plane determined by two normal vectors in binocular vision.

In order to refine the laser plane and reconstruct the 3D intersection points between the measured object and the laser plane, we design an L target with three feature points **Q**_*i*,1_, **Q**_*i*,2_, **Q**_*i*,3_ in the *i*-th position. *i* = 1, 2, …, *n*. In Fig. 3, the laser plane intersects the L target with the three points. The projections of the three points captured by two cameras obey to the pinhole model^17^. Therefore, we have

8 $${{\bf{P}}}^{{\rm{\alpha }}}{{\bf{Q}}}_{i,j}^{{\rm{\alpha }}}={s}_{i,j}{{\bf{q}}}_{i,j}^{{\rm{\alpha }}}$$

9 $${{\bf{P}}}^{{\rm{\beta }}}{{\bf{Q}}}_{i,j}^{{\rm{\beta }}}={s}_{i,j}{{\bf{q}}}_{i,j}^{{\rm{\beta }}}$$

where ${{\bf{Q}}}_{i,j}^{{\rm{\alpha }}}$, ${{\bf{Q}}}_{i,j}^{{\rm{\beta }}}$ are the 3D points derived from two cameras and related to **Q**_*i*,1_, **Q**_*i*,2_, **Q**_*i*,3_. ${{\bf{q}}}_{i,j}^{{\rm{\alpha }}}={({x}_{i,j}^{{\rm{\alpha }}},{y}_{i,j}^{{\rm{\alpha }}})}^{{\rm{T}}}$, ${{\bf{q}}}_{i,j}^{{\rm{\beta }}}={({x}_{i,j}^{{\rm{\beta }}},{y}_{i,j}^{{\rm{\beta }}})}^{{\rm{T}}}$ are two projection points on the left and right cameras. *s*_*i*,*j*_ is a scale factor. *j* = 1, 2, 3.

The feature points on the laser plane satisfy^33^

10 $${({\boldsymbol{\phi }})}^{{\rm{T}}}{{\bf{Q}}}_{i,j}^{{\rm{\alpha }}}=0$$

11 $${({\boldsymbol{\phi }})}^{{\rm{T}}}{{\bf{Q}}}_{i,j}^{{\rm{\beta }}}=0$$

Stacking Eqs (8)–(11), the 3D points ${{\bf{Q}}}_{i,j}^{{\rm{\alpha }}}$, ${{\bf{Q}}}_{i,j}^{{\rm{\beta }}}$ are solved by the singular value decomposition (SVD) method^35^ and parameterized by

12 $${{\bf{Q}}}_{i,j}^{{\rm{\alpha }}}({\boldsymbol{\phi }})={f}_{i,j}^{{\rm{\alpha }}}({{\bf{R}}}_{i,j}^{{\rm{\alpha }}},{{\bf{S}}}_{i,j}^{{\rm{\alpha }}},{\boldsymbol{\phi }})$$

13 $${{\bf{Q}}}_{i,j}^{{\rm{\beta }}}({\boldsymbol{\phi }})={f}_{i,j}^{{\rm{\beta }}}({{\bf{R}}}_{i,j}^{{\rm{\beta }}},{{\bf{S}}}_{i,j}^{{\rm{\beta }}},{\boldsymbol{\phi }})$$

where

$$\begin{array}{rcl}{{\bf{R}}}_{i,j}^{{\rm{\alpha }}} & = & {[({p}_{11}^{{\rm{\alpha }}}-{p}_{31}^{{\rm{\alpha }}}{x}_{i,j}^{{\rm{\alpha }}}),({p}_{12}^{{\rm{\alpha }}}-{p}_{32}^{{\rm{\alpha }}}{x}_{i,j}^{{\rm{\alpha }}}),({p}_{13}^{{\rm{\alpha }}}-{p}_{33}^{{\rm{\alpha }}}{x}_{i,j}^{{\rm{\alpha }}}),({p}_{14}^{{\rm{\alpha }}}-{p}_{34}^{{\rm{\alpha }}}{x}_{i,j}^{{\rm{\alpha }}})]}^{{\rm{T}}},\\ {{\bf{S}}}_{i,j}^{{\rm{\alpha }}} & = & {[({p}_{21}^{{\rm{\alpha }}}-{p}_{31}^{{\rm{\alpha }}}{y}_{i,j}^{{\rm{\alpha }}}),({p}_{22}^{{\rm{\alpha }}}-{p}_{32}^{{\rm{\alpha }}}{y}_{i,j}^{{\rm{\alpha }}}),({p}_{23}^{{\rm{\alpha }}}-{p}_{33}^{{\rm{\alpha }}}{y}_{i,j}^{{\rm{\alpha }}}),({p}_{24}^{{\rm{\alpha }}}-{p}_{34}^{{\rm{\alpha }}}{y}_{i,j}^{{\rm{\alpha }}})]}^{{\rm{T}}},\\ {{\bf{R}}}_{i,j}^{{\rm{\beta }}} & = & {[({p}_{11}^{{\rm{\beta }}}-{p}_{31}^{{\rm{\beta }}}{x}_{i,j}^{{\rm{\beta }}}),({p}_{12}^{{\rm{\beta }}}-{p}_{32}^{{\rm{\beta }}}{x}_{i,j}^{{\rm{\beta }}}),({p}_{13}^{{\rm{\beta }}}-{p}_{33}^{{\rm{\beta }}}{x}_{i,j}^{{\rm{\beta }}}),({p}_{14}^{{\rm{\beta }}}-{p}_{34}^{{\rm{\beta }}}{x}_{i,j}^{{\rm{\beta }}})]}^{{\rm{T}}},\\ {{\bf{S}}}_{i,j}^{{\rm{\beta }}} & = & {[({p}_{21}^{{\rm{\beta }}}-{p}_{31}^{{\rm{\beta }}}{y}_{i,j}^{{\rm{\beta }}}),({p}_{22}^{{\rm{\beta }}}-{p}_{32}^{{\rm{\beta }}}{y}_{i,j}^{{\rm{\beta }}}),({p}_{23}^{{\rm{\beta }}}-{p}_{33}^{{\rm{\beta }}}{y}_{i,j}^{{\rm{\beta }}}),({p}_{24}^{{\rm{\beta }}}-{p}_{34}^{{\rm{\beta }}}{y}_{i,j}^{{\rm{\beta }}})]}^{{\rm{T}}}.\end{array}$$

As the 3D points ${{\bf{Q}}}_{i,j}^{{\rm{\alpha }}}({\boldsymbol{\phi }})$, ${{\bf{Q}}}_{i,j}^{{\rm{\beta }}}({\boldsymbol{\phi }})$ correspond to the feature point **Q**_*i*,*j*_, **Q**_*i*,*j*_ is balanced by ${{\bf{Q}}}_{i,j}^{{\rm{\alpha }}}({\boldsymbol{\phi }})$, ${{\bf{Q}}}_{i,j}^{{\rm{\beta }}}({\boldsymbol{\phi }})$ and parameterized by

14 $${{\bf{Q}}}_{i,j}({\boldsymbol{\phi }})=1/2[{{\bf{Q}}}_{i,j}^{{\rm{\alpha }}}({\boldsymbol{\phi }})+{{\bf{Q}}}_{i,j}^{{\rm{\beta }}}({\boldsymbol{\phi }})]$$

As the three 3D points on the L target generated two orthogonal vectors and the norms of the vectors are known, the orthogonality and norms of the vectors can be considered as two objects to optimize the laser plane. The object is given by

15 $$\begin{array}{rcl}\{\tilde{{\boldsymbol{\phi }}}\} & = & {\rm{\arg }}\,{\rm{\min }}\,\sum _{i=1}^{n}\{|\Vert {{\bf{Q}}}_{i,0}({\boldsymbol{\phi }})-{{\bf{Q}}}_{i,1}({\boldsymbol{\phi }})\Vert -{d}_{1}|\\  &  & +\,|\Vert {{\bf{Q}}}_{i,0}({\boldsymbol{\phi }})-{{\bf{Q}}}_{i,2}({\boldsymbol{\phi }})\Vert -{d}_{2}|\\  &  & +\,|\arccos \tfrac{({{\bf{Q}}}_{i,0}({\boldsymbol{\phi }})-{{\bf{Q}}}_{i,1}({\boldsymbol{\phi }}))({{\bf{Q}}}_{i,0}({\boldsymbol{\phi }})-{{\bf{Q}}}_{i,2}({\boldsymbol{\phi }}))}{\Vert ({{\bf{Q}}}_{i,0}({\boldsymbol{\phi }})-{{\bf{Q}}}_{i,1}({\boldsymbol{\phi }}))({{\bf{Q}}}_{i,0}({\boldsymbol{\phi }})-{{\bf{Q}}}_{i,2}({\boldsymbol{\phi }}))\Vert }-{\theta }|\}\end{array}$$

where $\tilde{{\boldsymbol{\phi }}}$ is the optimized laser plane of Eq. (15). *d*_1_, *d*_2_ are norms of the two vectors on the L target. *θ* is the angle between the two vectors.

As the orthogonality is scaled by degree and the norms of the vectors are scaled by millimeter, it is necessary to balance the two different objects. Consequently, the two objects in Eq. (15) are enhanced and normalized by

16 $$\begin{array}{rcl}\{\hat{{\boldsymbol{\phi }}}\} & = & {\rm{\arg }}\,{\rm{\min }}\,\sum _{i=1}^{n}\{|\Vert {{\bf{Q}}}_{i,0}({\boldsymbol{\phi }})-{{\bf{Q}}}_{i,1}({\boldsymbol{\phi }})\Vert -{d}_{1}|/{d}_{1}\\  &  & +\,|\Vert {{\bf{Q}}}_{i,0}({\boldsymbol{\phi }})-{{\bf{Q}}}_{i,2}({\boldsymbol{\phi }})\Vert -{d}_{2}|/{d}_{2}\\  &  & +\,|\arccos \tfrac{({{\bf{Q}}}_{i,0}({\boldsymbol{\phi }})-{{\bf{Q}}}_{i,1}({\boldsymbol{\phi }}))({{\bf{Q}}}_{i,0}({\boldsymbol{\phi }})-{{\bf{Q}}}_{i,2}({\boldsymbol{\phi }}))}{\Vert ({{\bf{Q}}}_{i,0}({\boldsymbol{\phi }})-{{\bf{Q}}}_{i,1}({\boldsymbol{\phi }}))({{\bf{Q}}}_{i,0}({\boldsymbol{\phi }})-{{\bf{Q}}}_{i,2}({\boldsymbol{\phi }}))\Vert }-{\theta }|/\theta \}\end{array}$$

where $\hat{{\boldsymbol{\phi }}}$ is the optimized laser plane of Eq. (16).

**Figure 4:**
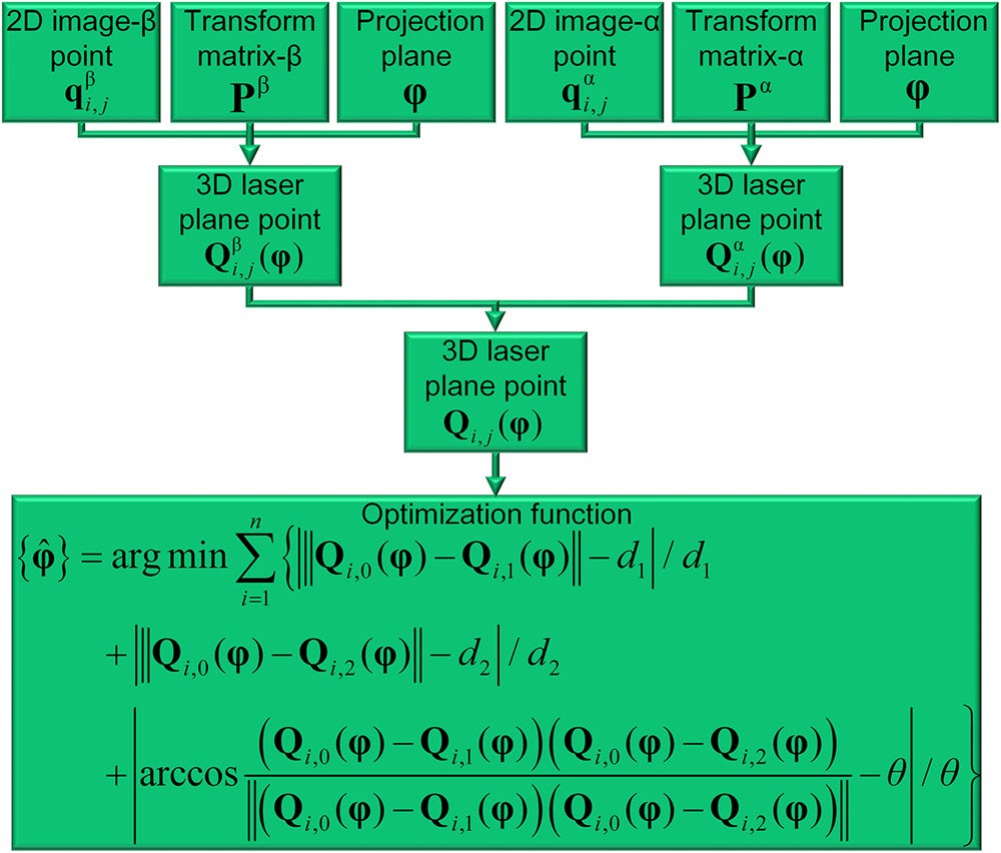
Optimization process of the laser plane using the binocular active vision with normalization object of vector orthogonality.

The optimization process of the laser plane in the binocular active vision is explained in Fig. 4. The benefits of the normalization object of vector orthogonality can be observed in experiment results.

In profile reconstruction, the laser plane intersects the measured object with 3D points **Q**. The epipolar geometry is employed to realize the matching task of the 2D points of the left image and the right image^36^. The corresponding point is considered as the nearest point to the epipolar line in the candidate set of laser points. The 3D points **Q** is located on the optimized laser plane $\hat{{\boldsymbol{\phi }}}$ and projected to the cameras^17,33^, then

17 $${(\hat{{\boldsymbol{\phi }}})}^{{\rm{T}}}{\bf{Q}}=0$$

18 $$({{\bf{P}}}^{{\rm{\alpha }}}){\bf{Q}}=s{{\bf{q}}}^{{\rm{\alpha }}}$$

19 $$({{\bf{P}}}^{{\rm{\beta }}}){\bf{Q}}=s{{\bf{q}}}^{{\rm{\beta }}}$$

where ${{\bf{q}}}^{{\rm{\alpha }}}={({x}^{{\rm{\alpha }}},{y}^{{\rm{\alpha }}})}^{{\rm{T}}}$, ${{\bf{q}}}^{{\rm{\beta }}}={({x}^{{\rm{\beta }}},{y}^{{\rm{\beta }}})}^{{\rm{T}}}$ are the projection points on two cameras. Eqs (17)–(19) can be rewritten by

20 $${[{\hat{{\boldsymbol{\phi }}}}^{{\rm{T}}}{({{\bf{R}}}^{\alpha })}^{{\rm{T}}}{({{\bf{S}}}^{\alpha })}^{{\rm{T}}}{({{\bf{S}}}^{\alpha })}^{{\rm{T}}}{({{\bf{S}}}^{{\rm{\beta }}})}^{{\rm{T}}}]}^{{\rm{T}}}{\bf{Q}}={\bf{0}}$$

where

$$\begin{array}{rcl}{{\bf{R}}}^{{\rm{\alpha }}} & = & {[({p}_{11}^{{\rm{\alpha }}}-{p}_{31}^{{\rm{\alpha }}}{x}^{{\rm{\alpha }}}),({p}_{12}^{{\rm{\alpha }}}-{p}_{32}^{{\rm{\alpha }}}{x}^{{\rm{\alpha }}}),({p}_{13}^{{\rm{\alpha }}}-{p}_{33}^{{\rm{\alpha }}}{x}^{{\rm{\alpha }}}),({p}_{14}^{{\rm{\alpha }}}-{p}_{34}^{{\rm{\alpha }}}{x}^{{\rm{\alpha }}})]}^{{\rm{T}}},\\ {{\bf{S}}}^{{\rm{\alpha }}} & = & {[({p}_{21}^{{\rm{\alpha }}}-{p}_{31}^{{\rm{\alpha }}}{y}^{{\rm{\alpha }}}),({p}_{22}^{{\rm{\alpha }}}-{p}_{32}^{{\rm{\alpha }}}{y}^{{\rm{\alpha }}}),({p}_{23}^{{\rm{\alpha }}}-{p}_{33}^{{\rm{\alpha }}}{y}^{{\rm{\alpha }}}),({p}_{24}^{{\rm{\alpha }}}-{p}_{34}^{{\rm{\alpha }}}{y}^{{\rm{\alpha }}})]}^{{\rm{T}}},\\ {{\bf{R}}}^{{\rm{\beta }}} & = & {[({p}_{11}^{{\rm{\beta }}}-{p}_{31}^{{\rm{\beta }}}{x}^{{\rm{\beta }}}),({p}_{12}^{{\rm{\beta }}}-{p}_{32}^{{\rm{\beta }}}{x}^{{\rm{\beta }}}),({p}_{13}^{{\rm{\beta }}}-{p}_{33}^{{\rm{\beta }}}{x}^{{\rm{\beta }}}),({p}_{14}^{{\rm{\beta }}}-{p}_{34}^{{\rm{\beta }}}{x}^{{\rm{\beta }}})]}^{{\rm{T}}},\\ {{\bf{S}}}^{{\rm{\beta }}} & = & {[({p}_{21}^{{\rm{\beta }}}-{p}_{31}^{{\rm{\beta }}}{y}^{{\rm{\beta }}}),({p}_{22}^{{\rm{\beta }}}-{p}_{32}^{{\rm{\beta }}}{y}^{{\rm{\beta }}}),({p}_{23}^{{\rm{\beta }}}-{p}_{33}^{{\rm{\beta }}}{y}^{{\rm{\beta }}}),({p}_{24}^{{\rm{\beta }}}-{p}_{34}^{{\rm{\beta }}}{y}^{{\rm{\beta }}})]}^{{\rm{T}}}.\end{array}$$

The 3D point **Q** on the measured object is solved by the SVD method.

## Results

**Figure 5:**
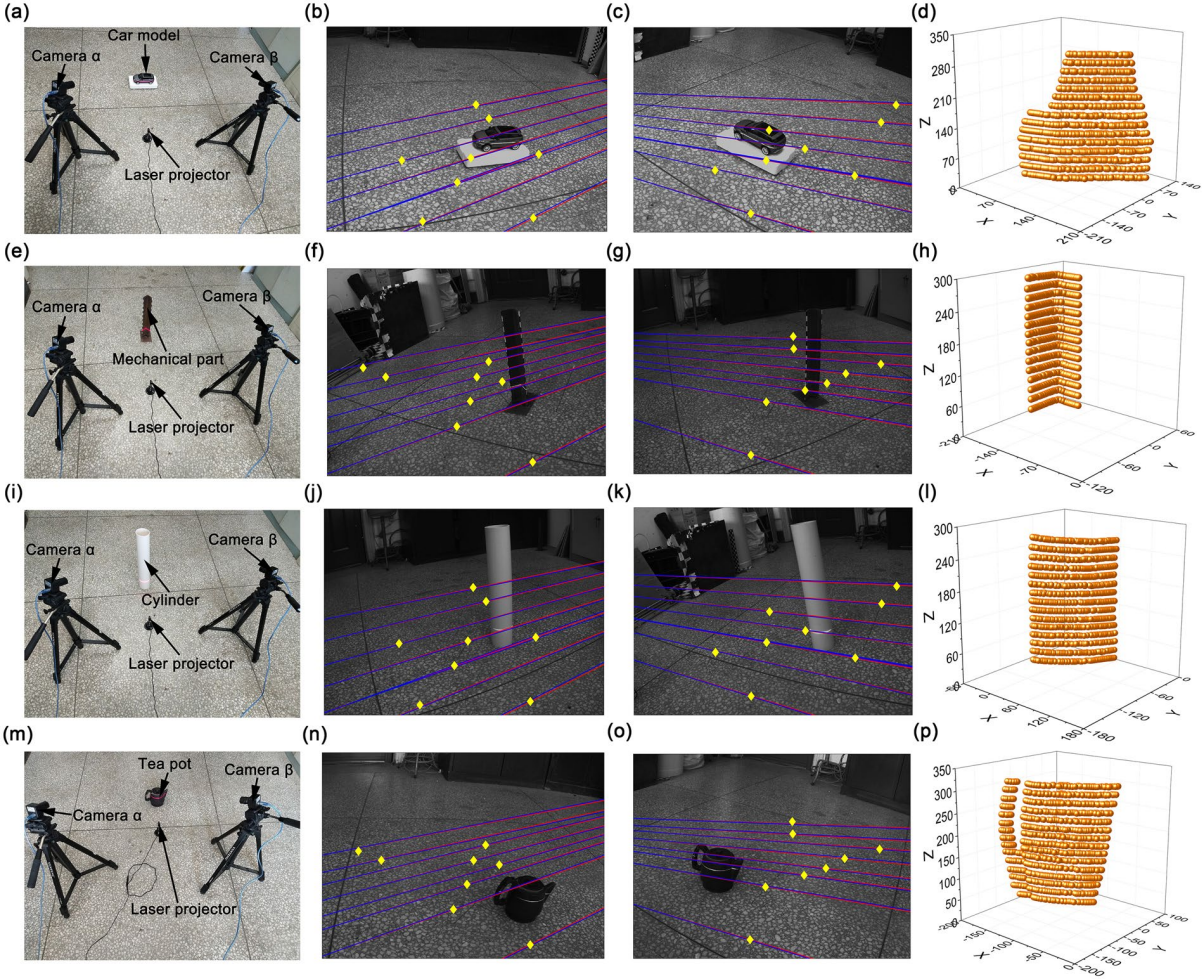
Experimental results of the profile measurement using the binocular active vision with the normalization object of vector orthogonality. (**a**, **e**, **i** and **m**) are the test system and the measured objects. (**b**, **f**, **j** and **n**) The images of the right camera, the feature points and epipolar lines. (**c**, **g**, **k** and **o**) The images of the left camera, the feature points and epipolar lines. (**d**, **h**, **l** and **p**) The reconstruction results of the measured objects.

**Figure 6:**
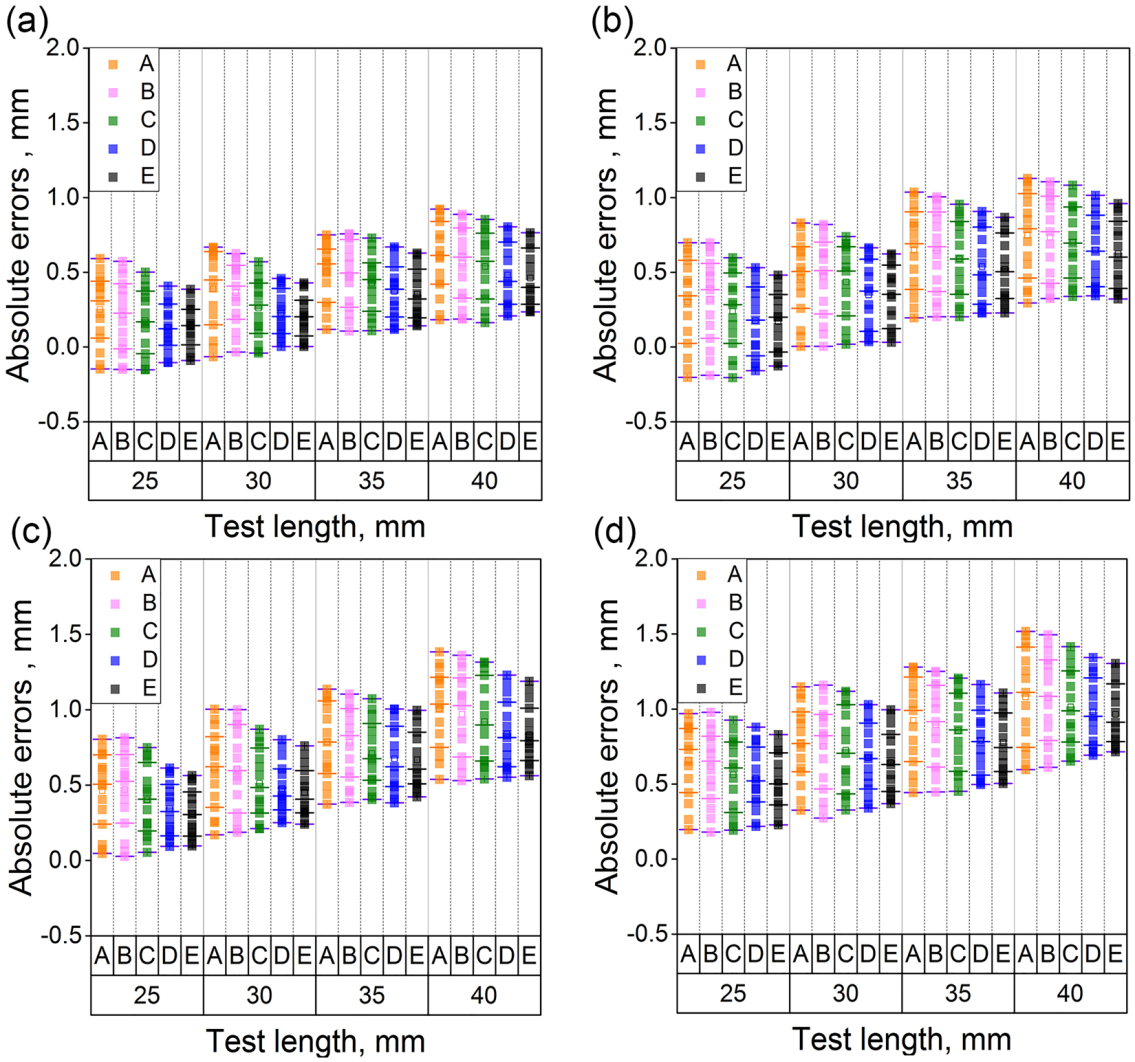
Absolute errors of the reconstructed test lengths based on A, B, C, D and E reconstruction methods. Method A is the binocular reconstruction vision. Method B is the monocular vision and a laser plane. Method C is the binocular vision and a laser plane without optimization. Method D is the binocular vision and a laser plane with the object of vector orthogonality. Method E is the binocular active vision with the normalization object of vector orthogonality. The error bar represent the distribution the reconstruction errors. Five line segments are located in every group of data. From the bottom line segment to the top line segment, 10%, 25%, 50%, 75%, 90% data are smaller than the related values of the line segments, respectively. (**a**–**d**) Are related to the measurement distances of 950 mm, 1000 mm, 1050 mm and 1100 mm, respectively.

The measured objects and a binocular-active-vision system, including a laser projector and two cameras, are shown in the first column of Fig. 5. 2048 × 1536 resolution is adopted in the experiments. The test codes are programmed by Matlab. The algorithms of the codes are based on the processes in Figs 2 and 4. Figure 2 describes the closed form solution of the laser plane. Figure 4 interprets the optimization process of the laser plane using the binocular active vision with normalization object of vector orthogonality. The profiles of four measured objects, a car model, a mechanical part, a cylinder and a tea pot, are sampled to show the validity of the method. The second and third columns in Fig. 5 provide the point-epipolar-line matching results of the left and right images. The profile measurement results are expressed in the last column of Fig. 5. Different from the traditional benchmark of the standard distance^25^, standard distances and standard angles are both chosen as the benchmarks to verify the proposed method. In the four groups of experiments, the normalization object of vector orthogonality is compared to other four reconstruction methods, which are the binocular vision, the monocular vision and a laser plane, the binocular vision and a laser plane without optimization, the binocular vision and a laser plane with the object of vector orthogonality. The standard lengths of this experiment are 25 mm, 30 mm, 35 mm and 40 mm while the standard angle is 90°. Figure 6 shows the errors of the reconstruction distances and standard distances. The measurement distances between the object and the line connecting two cameras are 950 mm, 1000 mm, 1050 mm and 1100 mm.

Figure 6(a) displays the experimental errors when the distance between the object and two cameras is 950 mm. The standard lengths of 25 mm, 30 mm, 35 mm and 40 mm are reconstructed by five methods, respectively. For the binocular vision method, the average reconstruction errors are 0.25 mm, 0.38 mm, 0.50 mm and 0.59 mm. The standard deviations are 0.22 mm, 0.24 mm, 0.22 mm and 0.24 mm. The average reconstruction errors using the monocular vision and the laser plane are 0.20 mm, 0.36 mm, 0.48 mm and 0.56 mm. The standard deviations are 0.20 mm, 0.22 mm, 0.22 mm and 0.24 mm. When the binocular vision and the laser plane without optimization is performed for reconstruction, the average reconstruction errors are 0.17 mm, 0.26 mm, 0.42 mm and 0.53 mm. The standard deviations are 0.20 mm, 0.20 mm, 0.22 mm and 0.24 mm. Based on the binocular vision and the laser plane with the object of vector orthogonality, the average errors are 0.13 mm, 0.22 mm, 0.37 mm and 0.48 mm. The standard deviations are 0.17 mm, 0.17 mm, 0.20 mm and 0.20 mm. For the binocular active vision with the normalization object of vector orthogonality, the average errors are 0.14 mm, 0.20 mm, 0.35 mm and 0.46 mm. The standard deviations are 0.14 mm, 0.14 mm, 0.17 mm and 0.20 mm. Tables 1 and 2 summarize the error means and standard deviations under the measurements of the standard lengths, 25 mm, 30 mm, 35 mm, 40 mm and the right angle. Moreover, Table 3 shows the uncertainties under the measurements of the standard lengths, 25 mm, 30 mm, 35 mm, 40 mm and the right angle.

**Table 1:** The error means under the measurements of the standard lengths, 25 mm, 30 mm, 35 mm, 40 mm and the right angle.

Measurement distance (mm)	Method	Error mean under different standard length and right angle							
		Length (mm)	Angle (°)	Length (mm)	Angle (°)	Length (mm)	Angle (°)	Length (mm)	Angle (°)
		25	90	30	90	35	90	40	90
950	A	0.25	0.43	0.38	0.57	0.50	0.89	0.59	1.01
	B	0.20	0.39	0.36	0.56	0.48	0.86	0.56	0.99
	C	0.17	0.30	0.26	0.43	0.42	0.76	0.53	0.82
	D	0.13	0.18	0.22	0.36	0.37	0.68	0.48	0.75
	E	0.13	0.14	0.20	0.32	0.35	0.62	0.46	0.72
1000	A	0.30	0.59	0.46	0.69	0.64	0.92	0.74	1.06
	B	0.31	0.54	0.47	0.66	0.64	0.92	0.73	1.05
	C	0.24	0.46	0.43	0.60	0.58	0.85	0.70	0.99
	D	0.17	0.39	0.34	0.54	0.53	0.78	0.64	0.96
	E	0.17	0.36	0.33	0.48	0.51	0.77	0.62	0.94
1050	A	0.46	0.65	0.59	0.90	0.78	1.20	1.00	1.38
	B	0.47	0.62	0.60	0.89	0.78	1.13	0.97	1.35
	C	0.40	0.53	0.51	0.71	0.70	1.06	0.92	1.27
	D	0.34	0.48	0.47	0.65	0.67	0.97	0.83	1.22
	E	0.31	0.45	0.45	0.60	0.66	0.96	0.83	1.22
1100	A	0.65	0.77	0.77	0.98	0.92	1.21	1.08	1.44
	B	0.61	0.71	0.75	0.98	0.89	1.17	1.07	1.45
	C	0.56	0.63	0.72	0.79	0.85	1.07	1.01	1.35
	D	0.53	0.58	0.67	0.73	0.79	1.01	0.98	1.24
	E	0.51	0.55	0.64	0.69	0.77	1.00	0.96	1.22

**Table 2:** The standard deviations under the measurements of the standard lengths, 25 mm, 30 mm, 35 mm, 40 mm and the right angle.

Measurement distance (mm)	Method	Standard deviation under different standard length and right angle							
		Length (mm)	Angle (°)	Length (mm)	Angle (°)	Length (mm)	Angle (°)	Length (mm)	Angle (°)
		25	90	30	90	35	90	40	90
950	A	0.22	0.40	0.24	0.40	0.22	0.45	0.24	0.45
	B	0.20	0.36	0.22	0.38	0.22	0.44	0.24	0.44
	C	0.20	0.30	0.20	0.32	0.22	0.37	0.24	0.38
	D	0.17	0.30	0.17	0.32	0.20	0.36	0.20	0.36
	E	0.14	0.29	0.14	0.30	0.17	0.32	0.20	0.33
1000	A	0.24	0.33	0.24	0.33	0.26	0.32	0.28	0.32
	B	0.24	0.28	0.26	0.30	0.26	0.33	0.28	0.32
	C	0.24	0.24	0.24	0.26	0.26	0.28	0.24	0.30
	D	0.22	0.24	0.22	0.24	0.24	0.25	0.24	0.27
	E	0.20	0.22	0.20	0.23	0.22	0.25	0.22	0.26
1050	A	0.24	0.30	0.26	0.33	0.26	0.36	0.28	0.36
	B	0.24	0.32	0.26	0.34	0.26	0.34	0.28	0.36
	C	0.22	0.24	0.22	0.28	0.22	0.28	0.26	0.30
	D	0.17	0.22	0.17	0.24	0.20	0.24	0.22	0.24
	E	0.17	0.20	0.17	0.20	0.20	0.22	0.20	0.22
1100	A	0.24	0.33	0.26	0.33	0.28	0.34	0.31	0.38
	B	0.24	0.32	0.28	0.32	0.26	0.33	0.28	0.37
	C	0.24	0.28	0.26	0.32	0.26	0.34	0.26	0.36
	D	0.20	0.24	0.22	0.26	0.22	0.30	0.22	0.30
	E	0.17	0.22	0.20	0.24	0.20	0.26	0.20	0.28

**Table 3:** The uncertainties under the measurements of the standard lengths, 25 mm, 30 mm, 35 mm, 40 mm and the right angle.

Measurement distance (mm)	Method	Uncertainty under different standard length and right angle							
		Length (mm)	Angle (°)	Length (mm)	Angle (°)	Length (mm)	Angle (°)	Length (mm)	Angle (°)
		25	90	30	90	35	90	40	90
950	A	0.22	0.27	0.25	0.29	0.22	0.30	0.26	0.31
	B	0.23	0.26	0.22	0.28	0.23	0.28	0.26	0.29
	C	0.20	0.24	0.19	0.26	0.20	0.27	0.25	0.27
	D	0.15	0.22	0.15	0.25	0.19	0.25	0.22	0.26
	E	0.14	0.20	0.14	0.22	0.18	0.22	0.20	0.24
1000	A	0.28	0.33	0.26	0.31	0.28	0.32	0.29	0.33
	B	0.28	0.30	0.27	0.30	0.27	0.29	0.28	0.31
	C	0.26	0.26	0.23	0.28	0.26	0.28	0.26	0.29
	D	0.24	0.25	0.23	0.26	0.24	0.27	0.26	0.29
	E	0.20	0.23	0.20	0.23	0.22	0.25	0.23	0.27
1050	A	0.26	0.32	0.28	0.33	0.27	0.34	0.29	0.34
	B	0.26	0.31	0.26	0.32	0.26	0.33	0.27	0.32
	C	0.25	0.28	0.27	0.30	0.25	0.33	0.27	0.31
	D	0.24	0.27	0.25	0.28	0.25	0.31	0.26	0.30
	E	0.22	0.25	0.22	0.27	0.23	0.28	0.25	0.28
1100	A	0.29	0.34	0.29	0.34	0.31	0.36	0.31	0.38
	B	0.28	0.30	0.29	0.33	0.30	0.35	0.30	0.36
	C	0.28	0.30	0.28	0.33	0.29	0.33	0.30	0.36
	D	0.27	0.28	0.27	0.30	0.29	0.32	0.28	0.35
	E	0.25	0.27	0.24	0.29	0.27	0.30	0.28	0.33

From Fig. 6(a–d), the average errors and the standard deviations increase when the distances between the object and the cameras change from 950 mm to 1100 mm. While the measurement distance is fixed, the average errors and the standard deviations drop down from method A to method E. Furthermore, the average errors and the standard deviations indicate an increasing trend when the standard length of the test grows up.

**Figure 7:**
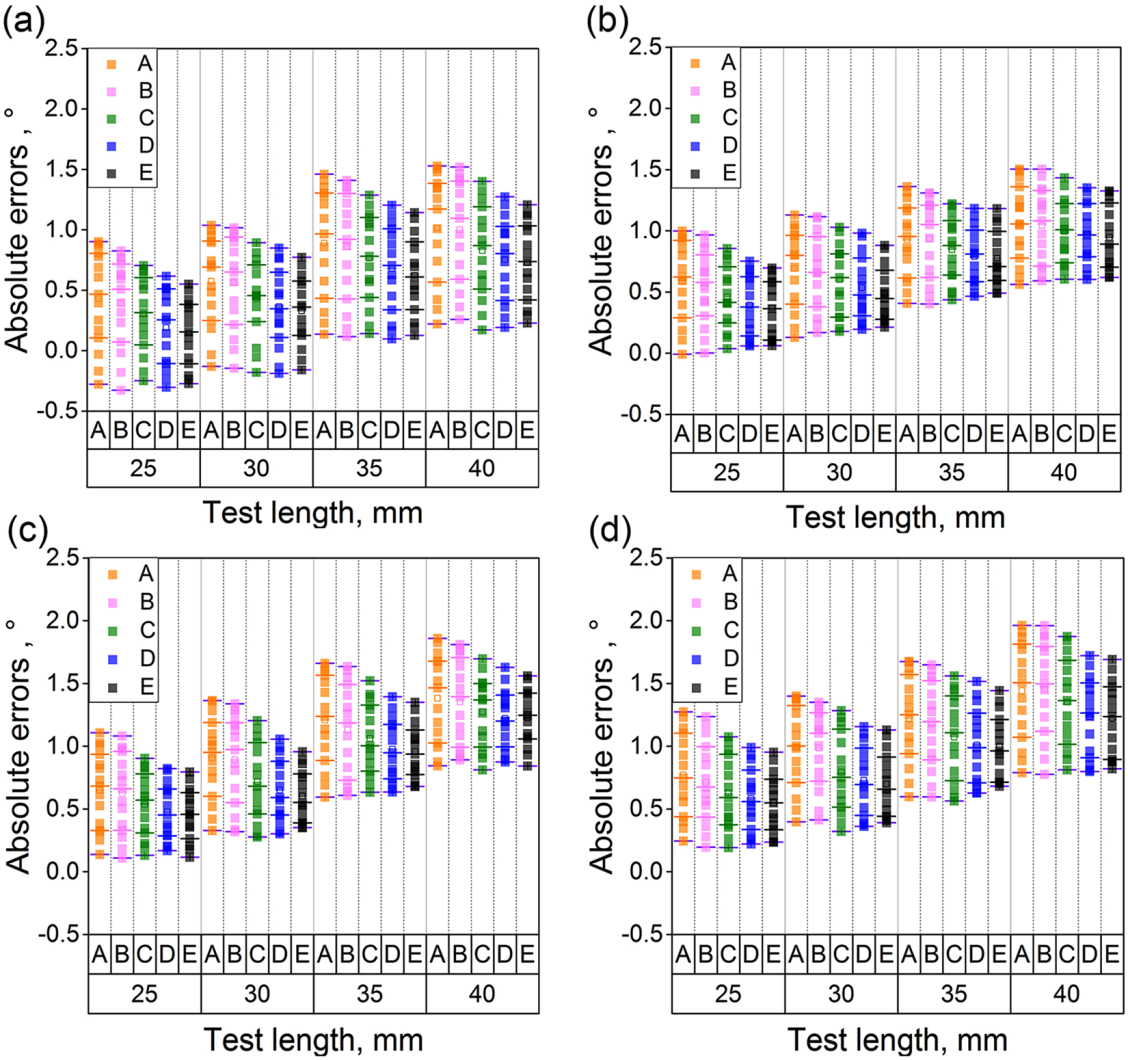
Absolute errors of the reconstructed test angles based on A, B, C, D and E reconstruction methods. Method A is the binocular reconstruction vision. Method B is the monocular vision and a laser plane. Method C is the binocular vision and a laser plane without optimization. Method D is the binocular vision and a laser plane with the object of vector orthogonality. Method E is the binocular active vision with the normalization object of vector orthogonality. The error bar represent the distribution the reconstruction errors. Five line segments are located in every group of data. From the bottom line segment to the top line segment, 10%, 25%, 50%, 75%, 90% data are smaller than the related values of the line segments, respectively. (**a**–**d**) Are related to the measurement distances of 950 mm, 1000 mm, 1050 mm and 1100 mm, respectively.

Figure 7 describes the angle errors in the test. The reconstructed standard angle is a right angle. Then the angle errors are obtained by comparing the reconstructed angles with the standard angle of 90°. In addition, the distances between the object and two cameras are also 950 mm, 1000 mm, 1050 mm and 1100 mm.

Figure 7(a) displays the experimental errors when the distance between the object and two cameras is 950 mm. For the different standard lengths, the average errors are 0.43°, 0.57°, 0.89° and 1.01° for the binocular reconstruction vision. The standard deviations are 0.40°, 0.40°, 0.45° and 0.45°. The method of the monocular vision and the laser plane expresses the reconstruction average errors of 0.39°, 0.56°, 0.86° and 0.99°. The standard deviations are 0.36°, 0.38°, 0.44° and 0.44°. For the third method, the average errors are 0.30°, 0.43°, 0.76° and 0.82°. The standard deviations are 0.30°, 0.32°, 0.37° and 0.38°. According to the method of the binocular vision and a laser plane with the object of vector orthogonality, the average errors are 0.18°, 0.36°, 0.68° and 0.75°. The standard deviations are 0.30°, 0.32°, 0.36° and 0.36°. For the last method, the average errors are 0.14°, 0.32°, 0.62° and 0.72°. The standard deviations are 0.29°, 0.30°, 0.32° and 0.33°. The experimental errors when the distances between the object and two cameras are 950 mm, 1000 mm, 1050 mm, 1100 mm are provided in Tables 1–3.

According to the results in Fig. 7(a–d), when the norm of the vector increases from 25 mm to 30 mm, 35 mm, 40 mm, the average angle errors and the standard deviations increase. The average errors and the standard deviations also grow up when the measurement distance varies from 950 mm to 1100 mm. Moreover, while the distance is fixed, the decreasing trend of the reconstruction angle errors is also observed from method A to method E. The errors of the binocular active vision with the normalization object of vector orthogonality are smaller than the ones of the binocular vision and a laser plane with the object of vector orthogonality. Therefore, the normalization object contributes more accurate results than other methods as it balances the length object and the angle object.

**Figure 8:**
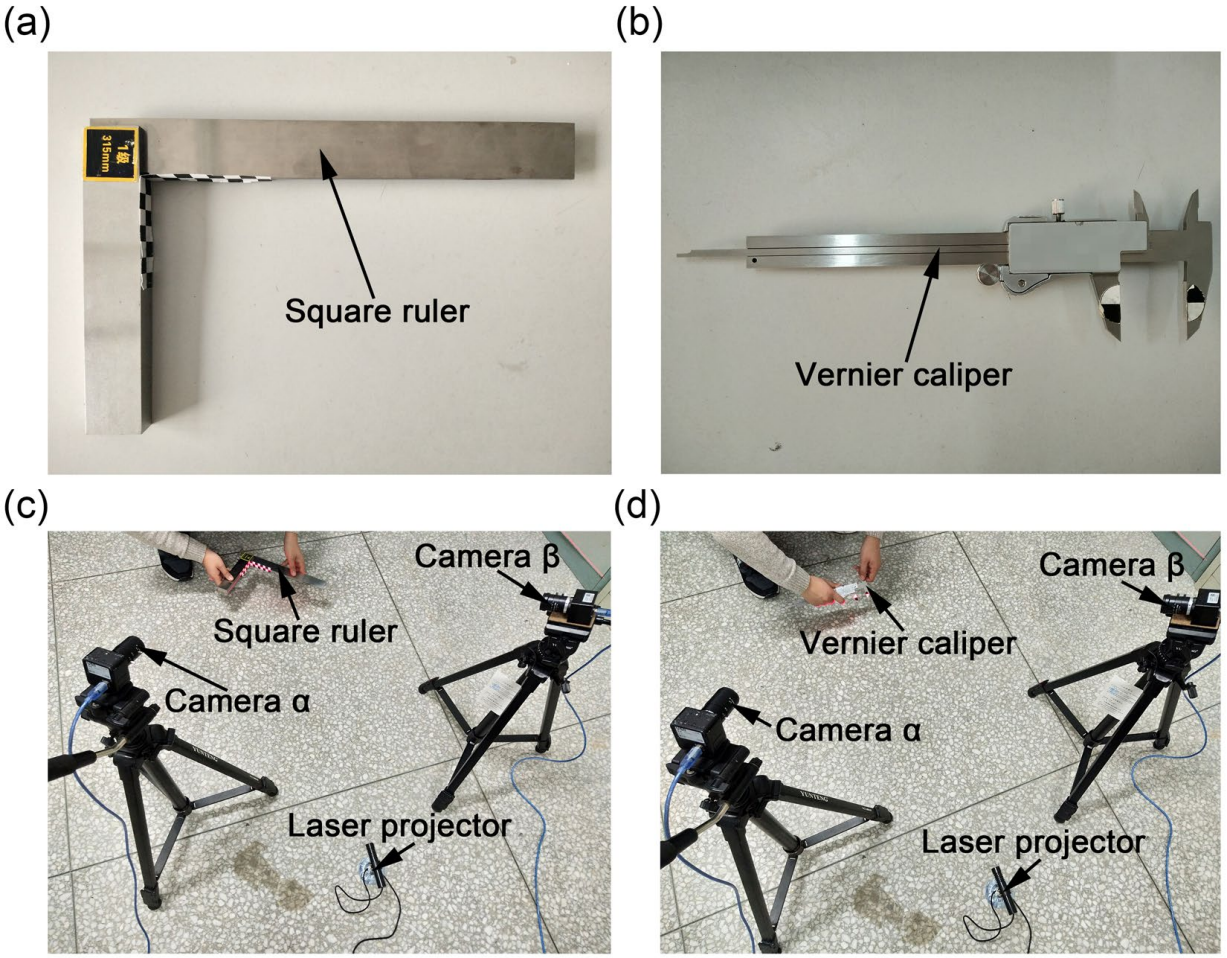
Experimental setups of the verification method of the profile measurement using the binocular active vision with the normalization object of vector orthogonality. (**a**) Is the square ruler for the absolute reference of the angle. (**b**) Is the vernier caliper for the absolute reference of the length. (**c**) Is the verification experiment with the square ruler. (**d**) Is the verification experiment with the vernier caliper.

In order to further investigate the accuracy of the approach, the first-level square ruler and the 0.01 mm vernier caliper are chosen as the quasi-ground-truths of the angle and the length, respectively. Figure 8 shows the experimental setups of the verification method of the profile measurement using the binocular active vision with the normalization object of vector orthogonality. The marks are attached to the two right sides of the square ruler. In order to recognize the standard length on the vernier caliper, two semi-circular marks are attached to the two outside large jaws of the vernier caliper, respectively. The vernier caliper provides the absolute reference lengths of 25 mm, 30 mm, 35 mm, and 40 mm, respectively. Table 4 shows the verification results of the measurement method with the quasi-ground-truths of the length and the angle. The experiment results show that the average reconstruction errors of the absolute lengths of the A, B, C, D and E methods are 0.63 mm, 0.60 mm, 0.55 mm, 0.51 mm and 0.49 mm, respectively. The average reconstruction errors of the absolute standard angle of the A, B, C, D and E methods are 0.91°, 0.87°, 0.78°, 0.71°, and 0.68°, respectively. In view of other methods, the binocular-active-vision method with the normalization object of the vector orthogonality achieves the higher accuracy for the reconstruction and is available for the object profile measurement and motion analysis.

**Table 4:** The verification results of the measurement method with the quasi-ground-truths of the length and the angle.

Measurement distance (mm)	Method	Error mean under different absolute length and right angle							
		Length (mm)	Angle (°)	Length (mm)	Angle (°)	Length (mm)	Angle (°)	Length (mm)	Angle (°)
		25	90	30	90	35	90	40	90
950	A	0.24	0.44	0.36	0.56	0.52	0.88	0.57	1.02
	B	0.21	0.39	0.32	0.56	0.47	0.84	0.54	0.98
	C	0.18	0.32	0.25	0.45	0.41	0.73	0.52	0.85
	D	0.15	0.17	0.22	0.34	0.35	0.65	0.47	0.77
	E	0.12	0.13	0.20	0.30	0.33	0.60	0.46	0.72
1000	A	0.29	0.57	0.45	0.70	0.65	0.94	0.76	1.04
	B	0.28	0.52	0.44	0.65	0.62	0.90	0.72	1.00
	C	0.23	0.44	0.43	0.60	0.56	0.85	0.68	0.96
	D	0.17	0.37	0.32	0.55	0.52	0.78	0.65	0.93
	E	0.16	0.36	0.31	0.47	0.49	0.76	0.62	0.91
1050	A	0.45	0.64	0.57	0.91	0.77	1.18	0.98	1.35
	B	0.42	0.61	0.53	0.89	0.74	1.13	0.95	1.31
	C	0.39	0.53	0.50	0.72	0.72	1.07	0.91	1.26
	D	0.36	0.47	0.45	0.64	0.68	0.99	0.84	1.22
	E	0.30	0.43	0.42	0.61	0.65	0.95	0.83	1.21
1100	A	0.65	0.75	0.79	0.96	0.93	1.22	1.07	1.41
	B	0.60	0.70	0.77	0.92	0.87	1.18	1.04	1.38
	C	0.55	0.63	0.74	0.77	0.84	1.09	0.99	1.34
	D	0.52	0.57	0.65	0.73	0.78	1.03	0.96	1.25
	E	0.51	0.53	0.65	0.69	0.75	1.01	0.93	1.22

## Summary

A profile measurement method adopting binocular active vision with the normalization object of vector orthogonality is presented in the paper. An L target is designed to construct two vectors in the view field of the cameras. The orthogonality of the vectors is achieved by the feature points on the target. Both length and angle of the vectors are considered in the optimization function. Furthermore, the length and angle of the vectors are normalized to balance the different optimization objects. Four methods are compared with the proposed method in the experiments. The experiments results show that the average errors of the reconstruction lengths using the five methods are 0.64 mm, 0.62 mm, 0.56 mm, 0.51 mm and 0.49 mm, respectively. The length errors of the method, compared with the other methods, are decreased by 23%, 20%, 14% and 4%, respectively. The average reconstruction errors of the five methods are 0.92°, 0.89°, 0.79°, 0.72° and 0.69°, respectively. The angle errors of the method, compared with the other methods, are reduced by 25%, 22%, 13% and 4%, respectively. Therefore, the binocular-active-vision method with normalization object of vector orthogonality balances the different objects of the angle and length and improves the accuracy of 3D objects reconstruction.

## Data Availability

The datasets generated during the current study are available from the corresponding author on reasonable request.

## References

[1] Kim D, Lee S (2013). Structured-light-based highly dense and robust 3D reconstruction. J. Opt. Soc. Am. A..

[2] Farid N, Hussein H, Bahrawi M (2015). Employing of diode lasers in speckle photography and application of FFT in measurements. Mapan-J. Metrol. Soc. I..

[3] Głowacz A, Głowacz Z (2017). Diagnosis of the three-phase induction motor using thermal imaging. Infrared Phys. Techn..

[4] Özgürün B, Tayyar DÖ, Agiş KÖ, Özcan M (2017). Three-dimensional image reconstruction of macroscopic objects from a single digital hologram using stereo disparity. Appl. Optics.

[5] Xu J, Xi N, Zhang C, Shi Q, Gregory J (2011). Real-time 3d shape inspection system of automotive parts based on structured light pattern. Opt. Laser Technol..

[6] Brosed FJ, Santolaria J, Aguilar JJ, Guillomía D (2012). Laser triangulation sensor and six axes anthropomorphic robot manipulator modelling for the measurement of complex geometry products. Robot. & Com-Int. Manuf..

[7] Ruiz J (2017). Breast density quantification using structured-light-based diffuse optical tomography simulations. Appl. Optics.

[8] Bakirman T, Gumusay MU, Reis HC (2017). Comparison of low cost 3D structured light scanners for face modeling. Appl. Optics.

[9] Geng J (2011). Structured-light 3d surface imaging: a tutorial. Adv. Opt. Photonics.

[10] Faugeras, O., Luong, Q. T. & Papadopoulo, T. *The geometry of multiple images - the laws that govern the formation of multiple images of a scene and some of their applications*. (DBLP, 2001).

[11] Wang X, Tieu K, Grimson WEL (2010). Correspondence-free activity analysis and scene modeling in multiple camera views. IEEE T. Pattern Anal..

[12] Wang, X., Tieu, K., & Grimson, W. E. L. Correspondence-free multi-camera activity analysis and scene modeling. *IEEE Conference on Computer Vision & Pattern Recognition* 1–8 (2008).

[13] Ren Z, Liao J, Cai L (2010). Three-dimensional measurement of small mechanical parts under a complicated background based on stereovision. Appl. Optics.

[14] Ambrosch K, Kubinger W (2010). Accurate hardware-based stereo vision. Comput. Vis. Image Und..

[15] Zabih, R. & Woodfill, J. Non-parametric local transforms for computing visual correspondence. *Proceedings of Computer Vision-ECCV* 151–158 (1994).

[16] Wang Z, Wu Z, Zhen X, Yang R, Xi J (2013). An onsite structure parameters calibration of large FOV binocular stereovision based on small-size 2D target. Optik.

[17] Zhang Z (2000). A flexible new technique for camera calibration. IEEE T. Pattern Anal..

[18] Luo Z, Zhang K, Wang Z, Zheng J, Chen Y (2017). 3D pose estimation of large and complicated workpieces based on binocular stereo vision. Appl. Optics.

[19] Li J (2017). Binocular vision measurement method for relative position and attitude based on dual-quaternion. J. Mod. Optic..

[20] Zhang X, Song Y, Yang Y, Pan H (2017). Stereo vision based autonomous robot calibration. Robot. Auton. Syst..

[21] Xu G, Yuan J, Li X, Su J (2017). Optimization reconstruction of projective point of laser line coordinated by orthogonal reference. Sci. Rep..

[22] Rodríguez J (2011). Laser imaging and approximation networks for calibration of three-dimensional vision. Opt. Laser Technol..

[23] Wang G, Hu Z, Wu F (2004). Implementation and experimental study on fast object modeling based on multiple structured stripes. Opt. Laser. Eng..

[24] Liu H, Su WH, Reichard K (2003). Calibration-based phase-shifting projected fringe profilometry for accurate absolute 3D surface profile measurement. Opt.Commun..

[25] Xu G, Yuan J, Li X, Su J (2017). 3D reconstruction of laser projective point with projection invariant generated from five points on 2d target. Sci. Rep..

[26] Chen X, Xi J, Jin Y (2009). Accurate calibration for a camera–projector measurement system based on structured light projection. Opt. Laser. Eng..

[27] Chen H (2016). Accurate calibration method for camera and projector in fringe patterns measurement system. Appl. Optics.

[28] Lelas M, Pribanić T (2016). Accurate stereo matching using pixel normalized cross correlation in time domain. Automatika.

[29] Zhang S (2008). Three-dimensional shape measurement using a structured light system with dual cameras. Opt. Eng..

[30] Vilaça JL, Fonseca JC, Pinho AM (2009). Calibration procedure for 3D measurement systems using two cameras and a laser line. Opt. Laser Technol..

[31] Jin ZS, Li HC, Li R, Sun YF, Gao HM (2019). 3D reconstruction of GMAW pool surface using composite sensor technology. Measurement.

[32] Abdel-Aziz YI, Karara HM, Hauck M (2015). Direct linear transformation from comparator coordinates into object space coordinates in close range photogrammetry. Photogramm. Eng. Rem. S..

[33] Hartley, R. & Zisserman, A. *Multiple View Geometry in Computer Vision* (Cambridge University Press, 2003).

[34] Wei Z (2010). Novel calibration method for a multi-sensor visual measurement system based on structured light. Opt. Eng..

[35] Horn, R. A. & Johnson, C. R. Matrix *Analysis* (Cambridge University Press, 2012).

[36] Linda, G. S. & George, C. S. *Computer Vision* (Prentice Hall, 2001).

